# Construction and Characterization of Fitting Equations for a New Wheat Straw Pulping Method

**DOI:** 10.3390/polym15244637

**Published:** 2023-12-07

**Authors:** Xiaoli Liang, Shan Wei, Yanpeng Xu, Liang Yin, Ruiming Wang, Piwu Li, Kaiquan Liu

**Affiliations:** 1 State Key Laboratory of Biobased Material and Green Papermaking (LBMP), Qilu University of Technology (Shandong Academy of Sciences), Jinan 250353, China; 10431211135@stu.qlu.edu.cn (X.L.); 10431211110@stu.qlu.edu.cn (Y.X.); wrm@qlu.edu.cn (R.W.); piwuli@qlu.edu.cn (P.L.); 2 Key Laboratory of Shandong Microbial Engineering, College of Bioengineering, Qilu University of Technology (Shandong Academy of Sciences), Jinan 250353, China; 1043118282@stu.qlu.edu.cn; 3 Gansu Engineering Technology Research Center for Microalgae, Hexi University, Zhangye 734000, China; yinl03@163.com

**Keywords:** wheat straw, lignin, Biopulping, xylanase, pectinase

## Abstract

The pretreatment of pulp with enzymes has been extensively studied in the laboratory. However, due to cost constraints, the application of enzymes in the pulp and paper industry is very limited. In this paper, an environment-friendly and efficient pulping method is proposed as an alternative to traditional pulping and papermaking methods. This new method overcomes the low efficiency and extreme pollution problems associated with traditional pulping methods. In addition, fitting equations for the new pulping method are constructed using data on enzyme treatments, which reflect the effect of enzymes and enable the realization of real-time control of the pulping process. The experimental results show that the efficiency of the pulping and papermaking process can be improved using biological enzymes, and the separation of cellulose can be facilitated using mixed enzymes, which have a better effect than single enzymes.

## 1. Introduction

According to relevant statistics, the total output of paper and paperboard in the global pulp and paper industry in 2022 is about 417 million tons, of which the total output of the United States is about 65.95 million tons, and the total output of China is about 124.32 million tons [1]. As the world’s largest developing country, China’s total volume of paper products ranks first in the world, but the per capita output of paper products is much lower than that of developed countries. According to statistics, in 2022, China’s pulp, paper and paper products industry achieved a total output of 283.91 million tons of pulp, paper, cardboard and paper products, with an average annual growth of 1.32%. Among them, the output of paper and paperboard was 124.25 million tons, an increase of 2.64% over the previous year. Pulp output was 85.87 million tons, an increase of 5.01% over the previous year. The output of paper products was 73.79 million tons, 4.65% less than the previous year [1]. In 2022, there are about 2500 paper and cardboard production enterprises in the country, and the national production of paper and cardboard is 124.25 million tons, an increase of 2.64% over the previous year. Over the previous year, consumption was 124.03 million tons, an increase of −1.94%, with a per capita annual consumption of 87.84 kg (1.412 billion people). According to the survey data of the China Paper Association, the total pulp production in 2022 will be 85.87 million tons, an increase of 5.01% over the previous year [2]. This includes 21.15 million tons of wood pulp, an increase of 16.92%; 59.14 million tons of waste pulp, an increase of 1.72% over the previous year; non-wood pulp was 5.58 million tons, an increase of 0.72% over the previous year [3]. In 2022, there will be 4727 paper products production enterprises above designated size in China, with a production capacity of 73.79 million tons, an increase of −4.65% over the previous year. The consumption was 68.97 million tons, an increase of −5.89% over the previous year. The import volume was 160,000 tons and the export volume was 4.98 million tons. From 2013 to 2022, the average annual growth rate of paper product production is 3.69%, and the average annual growth rate of consumption is 3.45% [2].

Although the demand for paper and paperboards has remained high in recent years, the shortage of raw materials has limited the development of the paper industry. The production capacity of pulp, particularly wood pulp, is insufficient to compensate for the high dependence on raw material imports. As a gramineous plant, wheat straw is an important source of biomass, which is grown globally. It is one of the three largest cereal crops in the world [4,5]. As agricultural waste, wheat straw employed in pulping not only reduces environmental pollution but also maximize straw resources [6]. Wheatgrass is an abundant crop by-product in China. With the continuous development of agricultural production science and technology and the improvement of wheat yields, the annual harvest of rice-wheatgrass has also increased [7]. Wheatgrass papermaking is an important aspect to promote the development of China’s papermaking industry. Of China’s non-wood pulp products, rice straw pulp and bamboo pulp make up the majority. In 2022, the domestic wheat straw pulp output was 1.5 million tons, an increase of 0.72% over the previous year, accounting for 26.9% of non-wood pulp output. The output of bamboo pulp was 2.46 million tons, accounting for 44.1% [2,8]. 

The chemical components of common deciduous wood were 58.61% cellulose, 22.71% pentosan, 17.04% lignin, 0.52% ash and 1.74% benzene-alcohol extract [8]. The chemical composition and content of wheat straw stem were: cellulose 47.09%, pentosan 32.28%, lignin 10.23%, ash 8.94% and benzene-alcohol extract 5.36% [9]. The average fiber length of wheatgrass was 1.32 mm, which was larger than that of broadleaf wood (1.03 mm) and smaller than that of coniferous wood (3.40 mm) [9]. Wheat straw has high cellulose content, short and fine fibers, low lignin molecular weight and wood ratio, and contains a large number of phenolic hydroxyl and ether bonds. It has strong lyophilic ability under alkaline environments, and can dissolve lignin at low temperatures to reduce energy waste [7].

Pulping mainly refers to the production process of using certain means, such as chemical reagents, papermaking machinery, or a combination of the two, to break down wood or other plant fiber raw materials, so that the fibers are dissociated and become unbleached color paste or bleached pulp [8]. The industry’s history dates back to ancient times, but its modernization began in the mid-19th century. After entering the 20th century, the pulp and paper industry began to widely use chemical pulping, mechanical pulping and chemical mechanical pulping [10]. With the continuous development of the pulp and paper industry, since entering the 21st century, the global pulp and paper industry has also begun to develop in the direction of green environmental protection, promoting the recycling of waste paper and green pulping technology to reduce the harm to the environment [9]. Traditional pulping methods include alkaline pulping and kraft pulping. To remove lignin and separate cellulose, these processes must be conducted under high-temperature conditions, requiring special equipment and long processing times [11]. Presently, sodium hydroxide pretreatment is the most widely used pulping method. This is because OH^−^ can act on the ether and ester bond of lignin to separate lignin and hemicellulose [12,13,14]. The solubility of lignin in different environments depends on different precursors or combinations of precursors [15]. Therefore, the enzyme activity should be considered when using chemical reagents to treat wheat straw.

The existing problems in the paper industry also include high energy consumption and environmental pressure. The chemical treatment of wheat straw will produce many harmful substances, including chlorophenol, dioxin, furan, fatty acid, resin acid and chlorolignin compounds. It has been confirmed that these chlorinated hydrocarbon organic pollutants are mainly produced by changes to the chemical structure of lignin during cooking and bleaching. These substances are harmful to the environment. Dioxins are easily produced when bleaching pulp with chlorine. These substances are detrimental to the environment [16]. Eventually, they will destroy the self-healing ability of the environment and cause irreversible harm.

Wheat straw pretreatment can reduce the obstinacy of the cell wall and increase the accessibility of enzymes to the carbohydrates in the cell wall [17]. Hydrothermal pretreatment changes the structure and molecular weight of lignin, which is mainly because of the breaking of chemical bonds and the formation of new carbon–carbon double bonds [18]. The hydrothermal pretreatment of lignin at high temperatures can exert a high-intensity inhibition effect on the enzyme, which is mainly because of the nonproduction adsorption and enzyme inactivation [17].

Studies have shown that the tensile index and energy absorption of the xylanase-treated wheat straw do not increase; however, its tear index decreases significantly [19]. Xylanase enhances the bleaching process and has a positive impact on pulp, paper and wastewater treatment, aiming to reduce the use of bleaching chemicals in the pulp refining process [6,20]. Xylanase can be employed in the pulping process, and its application is mainly facilitated by the existence of refractory lignin. The refractory property of lignin residues is partly attributed to the existence of xylan in hardwood kraft pulp, which is not easy to degrade and separate from the fiber [21,22]. The precipitated xylan forms a barrier on the fiber surface, which prevents the residual lignin from diffusing from the fiber wall. Moreover, hemicellulose combines with other fibrous substances and pectin through noncovalent and covalent bonds, indicating that the application of xylanase in the pulping process can improve the chemical extraction efficiency of lignin. Pectinase-treated pulp fiber has the characteristics of long fiber length, small fine fiber length, and high flexible fiber content, which are conducive for subsequent pulping.

In this paper, we propose a new strategy for improving the environmental impact of the pulping and papermaking processes. We attempt to produce pulp in a neutral environment. Through this approach, the production cost of the enzyme can be significantly reduced and the conditions for obtaining high-quality and low-cost paper can be achieved. In addition, pulping in a neutral environment reduces environmental pollution. In order to improve the pulping efficiency of the factory, we consider combining a certain index in the pulp after pulping with the degree of beater, exploring the law of the change of the index and the degree of beater, and describing the change of the index and the degree of beater through the fitting equation, so as to achieve the online control of biomechanical pulping [23,24].

## 2. Materials and Methods

### 2.1. Materials

The wheat straw used in this study was obtained from wheat fields in five different regions of China (Dezhou City, Shandong Province; Linfen City, Shanxi Province; Huaihua City, Anhui Province; Suqian City, Jiangsu Province; Puyang City, Henan Province). After the composition analysis of wheat straw, the wheat straw from Dezhou City, Shandong Province was used for the follow-up experiment. Before pulping, the raw materials were cleaned, cut and sampled with a length of approximately 1 cm, followed by drying under the sun.

The xylanase and pectinase used in the experiments were provided by Shandong Longkete Enzyme Preparation Co., Ltd. (Yishui, China). All the other reagents used were analytically pure.

### 2.2. Pretreatment

The dried wheat straw and water in the ratio of 1:8 were placed in an 80 °C water bath for 2 h for swelling treatment, and enzymes were added simultaneously. After 2 h, a 1.5% potassium hydroxide (KOH) solution was added. The mixture solution was left to stand for 1 h, after which the pH was adjusted to neutral with phosphoric acid.

### 2.3. Enzymolysis

Under neutral conditions, enzyme treatment was conducted at different temperatures (50–90 °C) for 1 h, after which the treated samples were ground using a refiner with revolutions of 3000 rpm. After refining, the enzyme was inactivated by placing the pulp in a water bath at 100 °C for 10 min, after which the samples were taken for analysis.

### 2.4. Analysis Method

#### 2.4.1. Composition Analysis of Wheat Straw

The contents of cellulose, hemicellulose and lignin in the wheat straw samples were determined according to the standard laboratory analysis procedure tp-510-42618 of the Renewable Energy Laboratory (NREL) [25]. The percentages of cellulose, hemicellulose and lignin in the samples were determined relative to the dry basis. The content of pectin was determined by carbazole colorimetry. The wheat straw was dried to a constant weight at (105 ± 2) °C, after which its water content was calculated. The wheat straw was placed in a muffle furnace and burned at (550 ± 10) °C for 2 h to determine its ash content (NREL/TP-510-42622) [26]. The protein content of the wheat straw was measured by the Kjeldahl method, according to NREL/TP-510-42625, and the nitrogen factor was 5.70 [27]. The fat content was determined by alkaline hydrolysis.

#### 2.4.2. Determination and Definition of the Enzyme Activity

The activities of xylanase and pectinase were determined using the standard 3,5-dinitrosalicylic acid method [28]. The enzyme activity in the enzyme treatment is defined as the amount of enzyme required to generate 1 μmol of the substrate in 1 min.

#### 2.4.3. Determination of the Main Detection Indexes

The reducing sugar content was determined by Fehling’s Reagent Titration method [29]. Three parallel determinations were carried out in each group. The content of soluble solids was measured by drying at 105 °C, and each experiment was carried out three times in parallel. The pulp’s Schober beating degree was measured using the IMT-DJD02 beating degree tester (Dongguan international material tester precision instrument Co., LTD, Dongguan, China); the data were kept to two decimal places, and each group of experiments was conducted three times in parallel.

#### 2.4.4. Establishment of the Fitting Equation

A Plackett–Burman test with Design-Expert 12.0 software was used to screen out three significant influencing factors, and the evaluation index was selected as the beating degree. The Box–Behnken response surface method was used to optimize the pulping process. MATLAB R2017a software was used to nonlinear fit the change of reducing sugar content and soluble solid content in the pulp after pulping and the change of beating degree.

#### 2.4.5. Scanning Electron Microscopy (SEM) Analysis

Using the Thermo Verios XHR SEM model scanning electron microscope (Thermo Fisher Scientific (China) Co., Ltd., Shanghai, China). The sample was freeze-dried to remove moisture. A conductive adhesive tape was glued to the sample table, on which the freeze-dried samples were dispersed. Due to the poor conductivity of the sample to be observed, it was necessary to spray gold.

The morphology of the enzyme treated wheat straw was analyzed by SEM. The secondary electron resolution is 3.5 nm; acceleration voltage 200 V–30 KV; and magnification 20–200,000×.

#### 2.4.6. Fourier Transform Infrared Analysis (FT-IR)

Using the BRUKER TENSOR Fourier infrared spectrometer (Bruker Corporation, Billerica, MA, USA). The samples were ground after freeze-drying and analyzed using a FT-IR. The wavelength absorption range was 400–4000 cm^−1^, the resolution was 0.5 cm^−1^, and the signal-to-noise ratio was 4000:1.

#### 2.4.7. The X-ray Diffraction (XRD) Sample Preparation and Crystallinity Analysis

Using the Rigaku SmartLab SE X-ray diffractometer (Rigaku Corporation, Tokyo, Japan), cellulose of wheat straw was isolated following the method described in other previous research. The freeze-dried material is ground into fine particles and subsequently put in the sample tank. Diffraction patterns were obtained using an X-ray powder diffractometer, and a copper target was used; Cu Kα generates X-rays under the conditions of an acceleration voltage of 40 kV and a current of 40 mA. The scanning angle range was 5–50°. After scanning, the crystallinity of the sample was determined.

The calculation formula for the crystallinity index is as follows:

$$CrI\;(\%)=\frac{I_{200}-I_{\mathrm{am}}}{I_{200}}\times 100\%$$

where *I*_200_ is the intensity of the crystalline peak at the maximum 2θ value between 22° and 23° for cellulose I (between 18° and 22° for cellulose II), and *I*_am_ is the intensity at the minimum 2θ between 18° and 19° for cellulose I (between 13° and 15° for cellulose II) [30,31,32]. 

## 3. Results and Discussion

### 3.1. Components of Wheat Straw

The main components of wheat straw from five different sources were quantitatively determined, and the results are shown in Table 1. The results showed that the cellulose content of wheat straw in Dezhou City of Shandong Province and Huaihua City of Anhui Province were 47.09% and 47.15%, respectively. The lignin content and pectin content of the former were 2.52% and 0.51% lower than the latter, respectively. Therefore, the wheat straw from Dezhou City, Shandong Province, was selected for the experiment. Table 1 shows a comparison of the compositions of the wheat straws obtained from different producing areas.

### 3.2. Enzyme Activity Determination at Different Temperatures and pH Values

**Figure 1:**
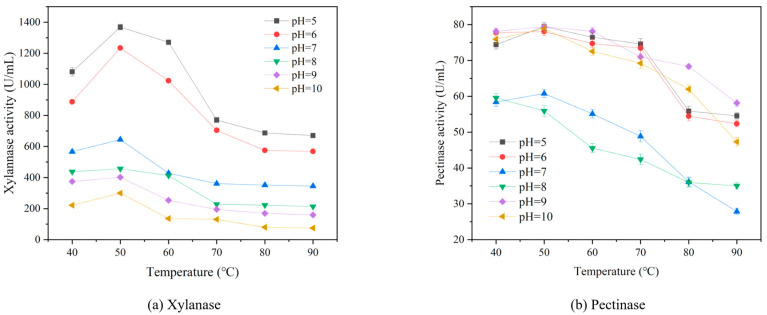
The activities of xylanase and pectinase at different temperatures (40–90 °C) and pH (5–10).

The actual enzyme activities of xylanase and pectinase were measured at 40–90 °C and pH 5.0–10.0, separately. Figure 1 shows that when the temperature was fixed, the actual enzyme activity of pectinase was relatively low at pH 7.0 and 8.0. A possible reason is that the types of enzymes commercially sourced are different; thus, they show different pH preferences. When the temperature is fixed, the enzyme activity of xylanase decreases as the pH increases. The optimum temperature of xylanase and pectinase is 50 °C. 

### 3.3. Effect of Enzyme Treatment on the Reducing Sugar and Soluble Solid Contents

**Figure 2:**
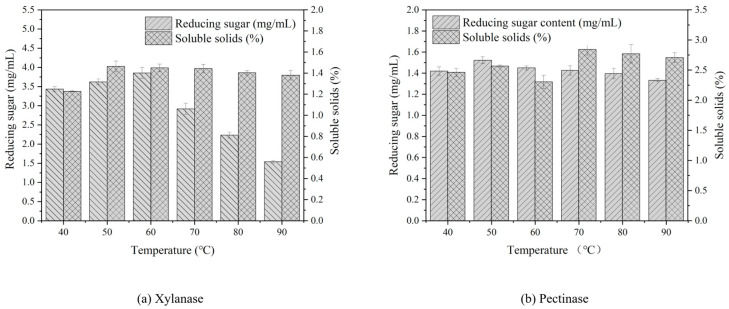
The reducing sugar and soluble solid contents in different enzyme treatments.

Figure 2 shows that the optimal temperature for xylanase to act in the pulping process is approximately 50–60 °C. The reducing sugar content was the highest at 60 °C, reaching 3.69 mg/mL, whereas the soluble solid content was the highest at 50 °C, which was 1.46%. The reducing sugar content increased notably in the range of 40–60 °C, whereas it exhibited a downward trend in the range of 60–90 °C. The soluble solid content increased from 40 °C to 50 °C and subsequently decreased from 50 °C to 90 °C. The optimal temperature of pectinase in the pulping process was 50 °C, at which point the reducing sugar content was the highest, reaching 1.52 mg/mL. The second highest content was observed at 60 °C. To facilitate the effect of pectinase in the pulping process, 70 °C was employed, at which point the soluble solid content was the highest (2.84%). However, it should be noted that the soluble solid content increased with the temperature from 40 °C to 50 °C. When the temperature increased to 60 °C, the soluble solid content decreased. The soluble solid content increased between 60 °C and 80 °C. A possible reason for this phenomenon is that the soluble solids were affected by the superposition of the optimal temperature and the enzyme. After 60 °C, the effect of the temperature on the soluble solids was greater than that of the enzyme.

### 3.4. Box–Behnken Response Surface Methodology

This method evaluates the three significant factors in the Plackett–Burman experiment while keeping the other factors at their central level. The range and level of the investigated variables are shown in Table 2a. The Box–Behnken design has three factors and three levels, including four replications of the center point, which are used to fit the second-order response surface. A polynomial quadratic equation is obtained to determine the influence of each variable on the response. The design and results of the trial scheme are shown in Table 2b, and the analysis of variance is shown in Table 2c.

The binomial fitting equation obtained by statistical analysis is as follows:

$$\begin{array}{cc} Y= & 37.62-6.20\times A-2.94\times B-3.95\times C+0.1450\times AB\\ & -0.1050\times BC-0.7255\times A^{2}+0.1295\times B^{2}-0.1005\times C^{2} \end{array}$$

The *p* value of the fitting equation is less than 0.05, which indicates that the binomial model has reached a significant degree, the fitting of the model is good and the response value can be detected.

### 3.5. Establishment of the Fitting Equation

(1) Establishment of the fitting equation between the beating degree and reducing sugar content.

Matlab R2017a was used to fit the reducing sugar content and beating degree, and the fitting equation of the Fourier transform distribution was obtained (Formula (1)). The values and confidence limits of each constant at 95% confidence are shown in Table S1.

(1) $$\begin{array}{cc} f(x)= & 822.8+1553*\cos(x*5.464)+916.2*\sin(x*5.464)\\ & +584.3*\cos(2*x*5.464)+2019*\sin(2*x*5.464)\\ & -1162*\cos(3*x*5.464)+1696*\sin(3*x*5.464)\\ & -1613*\cos(4*x*5.464)+51.8*\sin(4*x*5.464)\\ & -621.1*\cos(5*x*5.464)-762*\sin(5*x*5.464)\\ & +102*\cos(6*x*5.464)-417.6*\sin(6*x*5.464)\\ & +93.34*\cos(7*x*5.464)-51.8*\sin(7*x*5.464) \end{array}$$

(2) Establishment of the fitting equation between the beating degree and the soluble solids.

Matlab R2017a was used to fit the values of the soluble solid content and beating degree, and the fitting equation of the Fourier transform distribution was obtained (Formula (2)). The values and confidence limits of each constant at 95% confidence are shown in Table S2.

(2) $$\begin{array}{cc} f(x)= & -3387+1894*\cos(x*8.213)+6240*\sin(x*8.213)\\ & +4649*\cos(2*x*8.213)-3242*\sin(2*x*8.213)\\ & -3667*\cos(3*x*8.213)-2506*\sin(3*x*8.213)\\ & -452*\cos(4*x*8.213)+2940*\sin(4*x*8.213)\\ & +1461*\cos(5*x*8.213)-581.9*\sin(5*x*8.213)\\ & -501.9*\cos(6*x*8.213)-327.9*\sin(6*x*8.213)\\ & +24.22*\cos(7*x*8.213)+129.3*\sin(7*x*8.213) \end{array}$$

The goodness of fit of the obtained fitting equation was tested, and the results are shown in Table S3.

When the reducing sugar content was 2.2 mg/mL, the beating degree predicted according to Formula (1) was approximately 35.05°SR, and the error from the actual measured value was 1.26%; at this time, the soluble solid content was 1.64%. The beating degree predicted according to Formula (2) was approximately 35.57°SR, and the error from the actual value was 0.20%. Briefly, from the comparison of the goodness of fit of the above two equations, it was found that the fit equations of the degree of beating and soluble solid content are better than those of the degree of beating and reducing sugar content. Therefore, in the actual production process, one can choose to use the fitting equations of the beating degree and soluble solid content for the online control of the beating process.

### 3.6. Scanning Electron Microscopy Analysis of the Pulping Effect

Compared with the blank control without enzyme treatment, the use of xylanase increases the curvature of the fiber, and a small amount of fiber is separated; consequently, many fine fibers appear on the surface edge of the fiber. Compared with the xylanase-treated wheat straw case, the change in the fiber surface of the wheat straw treated with pectinase is noticeable, and more flakes of fiber are detached and attached to the whole fiber surface (as shown in Figure S1).

### 3.7. Infrared Analysis of the Pulping Effect

In the study of pulp properties, FT-IR is mainly used for the qualitative analysis of raw fiber, mainly through the analysis of the characteristic functional groups of fibrous substances to preliminarily determine the structural changes in the fibrous substances. Figure S2 shows a strong absorption band at 3408 cm^−1^, corresponding to the O–H bond stretching vibration of the untreated wheat straw, which indicates that the wheat straw fiber swells easily at this time. In addition, the absorption band at 1055 cm^−1^ may be assigned to the stretching vibration of the alkoxy group in the acetyl moiety and that of the carbon–oxygen double bond. After chemical and mechanical treatments, the absorption band intensities at 3408 and 1055 cm^−1^ decreased considerably, indicating that the structure of the hemicellulose was destroyed to some extent. The decrease in the absorption band intensities at 1512 and 1245 cm^−1^ also indicated that the structure of the lignin was destroyed considerably after treatment.

After the wheat straw was treated with different enzymes, FT-IR analysis was carried out on the pulp samples. The absorption band at 3418 cm^−1^, corresponding to the –OH stretching vibration in the pulp samples treated with xylanase and pectinase, was the strongest, indicating that more phenolic hydroxyl groups were produced and the hydration degree of the pulp was high. The formation of hydrogen bonds in cellulose reduces the hygroscopicity of fiber and paper. The strength of paper depends on the strength of the fiber itself and the bonding strength between the fibers. The beating process refines the fibers and exposes more hydroxyl groups on the surface. When the fibers are pulped in the paper machine and the paper is dried, hydrogen bonds are formed between the fibers, and the binding force is increased, resulting in a certain paper strength [33]. The absorption bands of the blank control at 1460 and 1423 cm^−1^ were significantly different from those after the enzyme treatment, indicating that enzyme treatment could change the benzene ring structure of lignin. The band at 1046 cm^−1^ corresponds to the symmetric stretching vibration of the C–O–C glycosidic bond. Compared with the blank control, the increase of absorption band intensity indicated the increase of cellulose content. Other functional groups and characteristic substances with notable structural changes are listed in Table 3.

### 3.8. X-ray Diffraction Analysis of the Pulping Effect

Compared with that of the untreated wheat straw, the peak intensities of the crystallization zone of the mechanically and chemically treated wheat straw decreased, and the crystallinity index decreased from 71.98% to 49.04%. According to Figure S3, the corresponding crystallinity indexes of the pulp treated by the enzymes are shown in Table 4. By comparison, it was found that the crystallinity of cellulose increased by varying degrees depending on the treatment, and the same conclusion has been reported elsewhere [34]. The crystallinity index of wheat straw increased by 1.19% after pectinase treatment, which was mainly because pectinase degraded the free poly galacturonic acid in pulp, reduced the anion waste in the system, reduced the proportion of amorphous area and increased the crystallization zone. The increase in the crystallinity of the pulp will increase its physical strength considerably. Notably, the effect of xylanase was the most significant. The crystallinity index of the wheat straw was the lowest after xylanase treatment: only 0.49% higher. The addition of xylanase degraded a part of the hemicellulose, which reduced the noncrystallized zone (mainly in the form of a noncrystalline zone) in the pulp, consequently increasing the crystallinity. Raw material and final pulp effect are shown in Figure S4.

## 4. Conclusions

In response to the problems of low yield and serious pollution in chemical pulping, we propose a biomechanical pulping method that combines xylanase and pectinase treatment under normal temperature and pressure conditions, which can reduce the dosage of drugs and the generation of pulping wastewater. The application of biological enzymes promotes the dissociation of cellulose, hemicellulose, lignin and other components in wheat straw, reduces the difficulty and improves the efficiency of pulping. At the same time, by establishing fitting equations for beating degree, reducing sugar and soluble solids, real-time monitoring of reducing sugar, soluble solids and other indicators can be achieved to achieve online control of important indicators such as beating degree in pulping, which is conducive to improving the automation level of straw pulping. In addition, some methods were used to characterize the pulping effect of the composite enzyme, and the results proved that the addition of complex enzymes had a significant positive influence on the pulping effect.

## Figures and Tables

**Table 1:** Comparison of the compositions of the wheat straws obtained from different producing areas.

Composition	Dezhou City, Shandong Province	Linfen City, Shanxi Province	Huaihua City, Anhui Province	Suqian City, Jiangsu Province	Puyang City, Henan Province
Cellulose (dry basis)	47.09%	34.47%	47.15%	36.06%	41.90%
Hemicellulose (dry basis)	32.28%	36.43%	35.06%	35.09%	31.67%
Lignin (dry basis)	10.23%	7.72%	12.75%	7.94%	8.51%
Pectin (dry basis)	1.45%	2.52%	1.96%	2.48%	1.95%
Ash (dry basis)	8.94%	4.89%	4.18%	5.47%	8.51%
Water content	9.11%	8.04%	9.01%	6.81%	7.17%
Protein	2.01%	2.86%	1.27%	2.51%	3.09%
crude fat	0.40%	0.20%	0.40%	0.20%	0.10%

**Table 2:** (a) Factors and levels of the Box–Behnken response surface experiment. (b) Box–Behnken response surface methodology test scheme design. (c) Analysis of variance table.

a						
Factor		Level				
		−1		0		1
KOH dosage (%)		1.5		2.0		2.5
Liquid solid ratio		7		8		9
revolutions (r)		2500		3000		3500
**b**						
Serial Number	KOH Dosage (%)	Liquid Solid Ratio		Revolutions (r)		Beating Degree (°SR)
1	2.0	8		3000		37.75
2	2.0	8		3000		37.33
3	2.5	7		3000		34.00
4	1.5	7		3000		46.00
5	1.5	8		2500		47.33
6	2.0	7		3500		36.50
7	1.5	9		3000		39.75
8	2.0	8		3000		37.50
9	2.0	7		2500		44.33
10	2.0	9		2500		38.75
11	2.0	8		3000		37.50
12	2.5	9		3000		28.33
13	2.0	8		3000		38.00
14	2.5	8		2500		34.25
15	1.5	8		3500		39.33
16	2.0	9		3500		30.75
17	2.5	8		3500		26.25
**c**						
Source	Sum of Squares	df	Mean Square	F Value	*p* Value Prob > F	
Model	503.42	9	55.94	313.47	<0.0001	significant
KOH dosage (%) (*A*)	307.27	1	307.27	1722	<0.0001	
Liquid solid ratio (*B*)	69.03	1	69.03	386.86	<0.0001	
revolutions (r) (*C*)	124.66	1	124.66	698.63	<0.0001	
*AB*	0.0841	1	0.0841	0.4713	0.5145	
*AC*	0	1	0	0	1	
*BC*	0.0441	1	0.0441	0.2471	0.6343	
*A* ^2^	2.22	1	2.22	12.42	0.0097	
*B* ^2^	0.0706	1	0.0706	0.3957	0.5493	
*C* ^2^	0.0425	1	0.0425	0.2383	0.6403	
Residual	1.25	7	0.1784			
Lack of fit	0.9749	3	0.325	4.74	0.0834	not significant
Pure error	0.2741	4	0.0685			
Cor total	504.67	16				

**Table 3:** FT-IR spectrum analysis after enzyme treatment.

Wavemunber (cm^−1^)	Corresponding Structure
3348–3408	O–H stretching vibration
2895–2902	C–H stretching vibration, CH_3_, CH_2_
1595–1597	Stretching vibration of benzene ring (lignin)
1460	CH_2_ deformation vibration, Carbon skeleton vibration of benzene ring
1423	CH_2_ shear vibration, CH_2_ bending vibration (lignin), Benzene ring vibration
1365–1371	C–H bending vibration
1327–1228	C–O–C stretching vibration (lignin phenol ether bond), Syringyl, Condensation guaiacol
1232–1234	Acetyl and hydroxyl vibration, Syringa type C=O stretching vibration
1034–1056	C–O–C glucoside bond symmetric stretching vibration

**Table 4:** Comparison of the cellulose crystallinity index under different treatment conditions.

	Blank Control	Xylanase	Pectinase	Xylanase + Pectinase
Crystallinity index (*CrI*%)	49.04	49.53	50.23	51.09

## Data Availability

Data are contained within the article and Supplementary Materials.

## Supplementary Materials

The following supporting information can be downloaded at: https://www.mdpi.com/article/10.3390/polym15244637/s1, Table S1. Values and confidence limits of the parameters in Formula 1 with 95% confidence; Table S2. Values and confidence limits of the parameters in Formula 2 with 95% confidence; Table S3. Goodness of fit test of the fitting equation; Figure S1. Scanning electron microscopy images of the blank control, xylanase, and pectinase treated wheat straw (from left to right); Figure S2. Fourier transform infrared image after the enzyme treatment; Figure S3. X-ray diffraction patterns of the pulp prepared by enzyme pretreatment; Figure S4. Starting material and final pulping effect.
