# Supplementary material for: Construction and Characterization of Fitting Equations for a New Wheat Straw Pulping Method
Source: Polymers (Basel). 2023 Dec 7;15(24):4637. doi: 10.3390/polym15244637 (PMC10748100; doi:10.3390/polym15244637)

Supporting Materials for polymers

Construction and Characterization of Fitting Equations for a New Wheat Straw Pulping Method

Xiaoli Liang [1,2,†], Shan Wei [2,†], Yanpeng Xu [1,2], Liang Yin [3], Ruiming Wang [1,2], Piwu Li [1,2] and Kaiquan Liu [1,2,*]

[1] State Key Laboratory of Biobased Material and Green Papermaking (LBMP), Qilu University of Technology (Shandong Academy of Sciences), Jinan 250353, China; 10431211135@stu.qlu.edu.cn (X.L.); 10431211110@stu.qlu.edu.cn (Y.X.); wrm@qlu.edu.cn (R.W.); piwuli@qlu.edu.cn (P.L.)

[2] Key Laboratory of Shandong Microbial Engineering, College of Bioengineering, QiLu University of Technology (Shandong Academy of Sciences), Jinan 250353, China; 1043118282@stu.qlu.edu.cn

[3] Gansu Engineering Technology Research Center for Microalgae, Hexi University, Zhangye 734000, China; yinl03@163.com

* Correspondence: liukq@qlu.edu.cn

† These authors contributed equally to this work.

Table S1. Values and confidence limits of the parameters in Formula 1 with 95% confidence.

Table S2. Values and confidence limits of the parameters in Formula 2 with 95% confidence.

Table S3. Goodness of fit test of the fitting equation.

Figure S1. Scanning electron microscopy images of the blank control, xylanase, and pectinase treated wheat straw (from left to right).

Figure S2. Fourier transform infrared image after the enzyme treatment.

Figure S3. X-ray diffraction patterns of the pulp prepared by enzyme pretreatment.

Figure S4. Starting material and final pulping effect.

Table S1. Values and confidence limits of the parameters in Formula 1 with 95% confidence.

Constant	Value	Lower confidence limit	Upper confidence limit
a_0	822.8	−1.618e + 838.8	1.618e + 838.8
a_1	1553	−3.084e + 1569	3.084e + 1569
b_1	916.2	−1.038e + 932.2	1.038e + 932.2
a_2	584.3	−3.049e + 600.3	3.049e + 600.3
b_2	2019	−2.254e + 2035	2.254e + 2035
a_3	− 1162	− 1.758e − 1146	1.758e − 1146
b_3	1696	− 4.266e + 1712	4.266e + 1712
a_4	− 1613	− 1.594e − 1597	1.594e − 1597
b_4	51.8	− 4.348e + 67.8	4.348e + 67.8
a_5	− 621.1	− 3.16e − 605.1	3.16e − 605.1
b_5	− 762	− 1.408e − 746	1.408e − 746
a_6	102	− 1.65e + 118	1.65e + 118
b_6	− 417.6	− 7.338e − 402.6	7.338e − 402.6
a_7	93.34	− 2.016e + 108.34	2.016e + 108.34
w	5.464	− 3.669e + 17.464	3.669e + 17.464

Table S2. Values and confidence limits of the parameters in Formula 2 with 95% confidence.

Constant	Value	Lower confidence limit	Upper confidence limit
A_0	− 3387	− 8e − 3372	− 8e − 3372
A_1	1894	− 1.319e + 1910	1.319e + 1910
B_1	6240	− 1.18e + 6256	1.18e + 6256
A_2	− 4649	− 1.301e + 4664	1.301e + 4664
B_2	− 3242	− 2.036e − 3226	− 2.036e − 3226
A_3	− 3667	− 1.82e − 3651	1.82e − 3651
B_3	− 2506	− 1.013e − 2490	1.013e − 2490
A_4	− 452	− 1.535e − 436	1.535e − 436
B_4	2940	− 8.116e + 2955	8.116e + 2955
A_5	1461	− 1.429e + 1476	1.429e + 1476
B_5	− 581.9	− 1.093e − 565.9	1.093e − 565.9
A_6	− 501.9	− 3.414e − 486.9	3.414e − 486.9
B_6	− 329.7	− 3.549e − 314.7	3.549e − 314.7
A_7	24.22	− 1.224e + 39.22	1.224e + 39.22
B_7	129.3	− 6.709e + 142.3	6.709e + 142.3
w	8.213	− 7.979e + 19.213	7.979e + 19.213

Table S3. Goodness of fit test of the fitting equation.

Fitting Equation 1		Fitting equation 2	
SSE	0.4063	SSE	0.3125
R^2	0.9989	R^2	0.9992
Adjusted R^2	0.9823	Adjusted R^2	0.9864
RMSE	0.6374	RMSE	0.559

Figure S1: Scanning electron microscopy images of the blank control, xylanase, and pectinase treated wheat straw (from left to right).

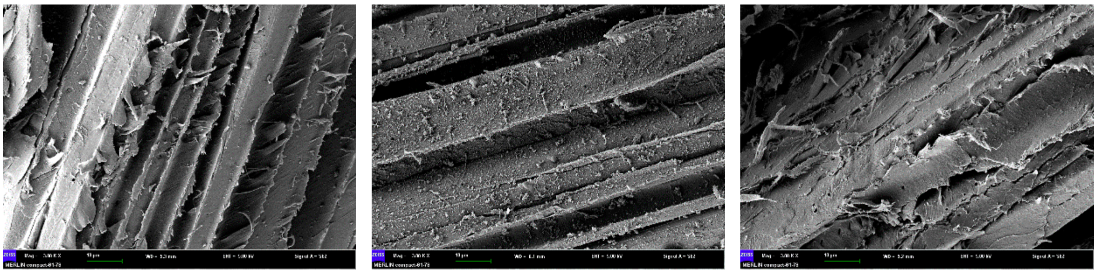

Figure S2: Fourier transform infrared image after the enzyme treatment.

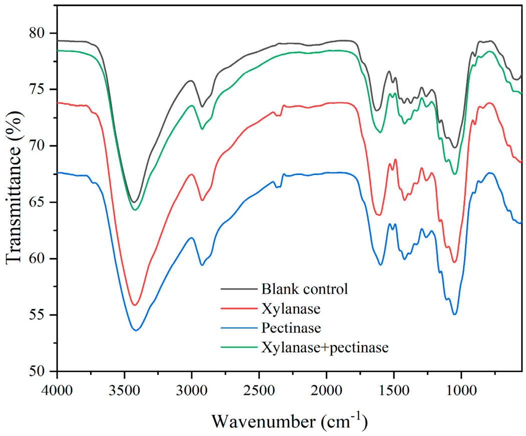

Figure S3: X-ray diffraction patterns of the pulp prepared by enzyme pretreatment.

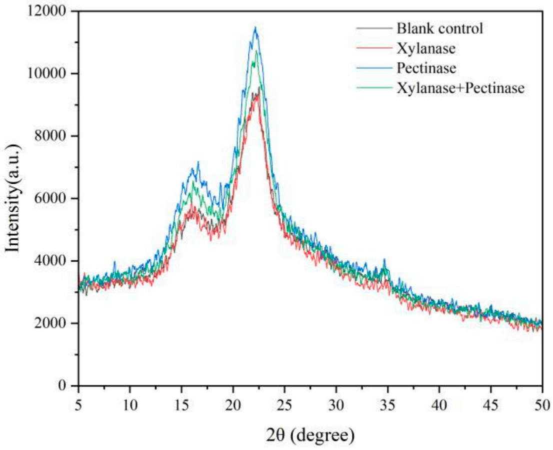

Figure S4: Starting material and final pulping effect.

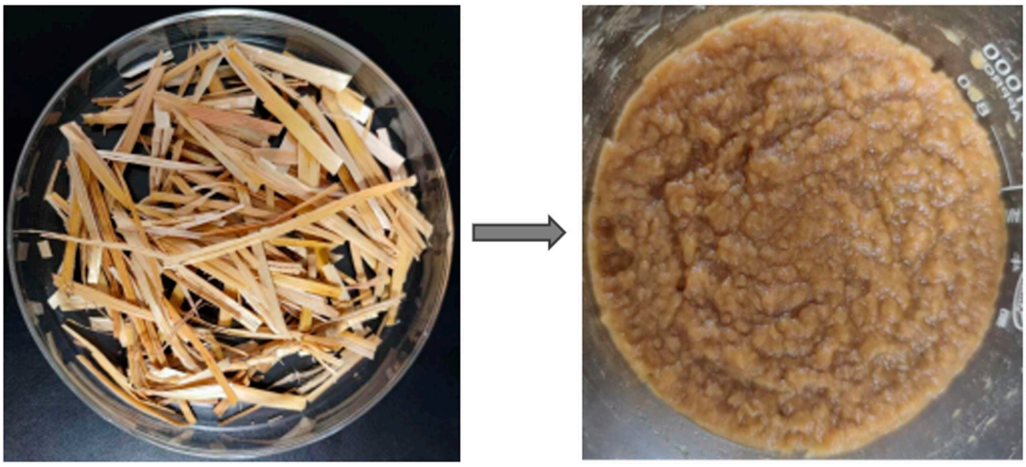

Supplement: Supplementary file 1 [file polymers-15-04637-s001.zip › polymers-2698638-supplementary.pdf]
